# The Influence of Oral Health on Comprehensive Health Outcomes in Critically Ill Patients

**DOI:** 10.7759/cureus.78605

**Published:** 2025-02-06

**Authors:** Basel N Alrawashdeh, Shadi A Hammadeen, Khaled N Hamadeneh, Hind M Almaaitah, Heba A Altarawneh

**Affiliations:** 1 Critical Care Medicine, Jordanian Royal Medical Services, Amman, JOR; 2 Anesthesiology and Critical Care, Jordanian Royal Medical Services, Amman, JOR; 3 Nephrology, Jordanian Royal Medical Services, Amman, JOR; 4 Dentistry, Jordanian Royal Medical Services, Amman, JOR

**Keywords:** clinical outcomes, critically ill patients, duration of stay, oral health status, overall mortality, ventilation status

## Abstract

Aims: The main purpose of the study was to find out how the oral health of critically ill patients affected the chances of bad outcomes such as longer hospital stays and death while taking into account possible confounders such as how often the critically ill patients used chlorhexidine mouthwash each week and their sequential organ failure assessment scores.

Methods: We conducted a retrospective observational study on 4,999 critically ill patients admitted to the King Hussein Medical Centre in Amman, Jordan, from January 2018 to May 2022. The patients were adults and the elderly, with a minimum of three consecutive days of admission. The study encompassed both mechanically ventilated and non-ventilated patients. We divided the cohort into two groups, Group I, based on favorable adverse outcomes, and Group II, based on unfavorable adverse outcomes. We examined the weekly administration of chlorhexidine mouthwash during critical care admissions as the principal variable. We conducted multiple logistic regression analyses to evaluate the correlation between weekly mouthwash usage and the likelihood of poorer outcomes while accounting for oral health conditions and the risk of critical illness.

Results: A retrospective study of 4,999 critically ill patients revealed that 2,370 patients (47.41%) achieved improved composite outcomes of interest (cOI), while 2,629 patients (52.26%) fell into the inferior cOI group. The overall mortality rate in the lower socioeconomic category was 1,920 patients (73%), compared to 709 patients (27%) in the survival rate. A study using multiple logistic regression showed a strong link between critically ill patients' oral health statuses (OHS), how often they used mouthwash each week (WMW), and their sequential organ failure assessment (SOFA) scores. The regression association for OHS relative to poorer cOI was positive, indicating a higher risk for poorer OHS compared to better OHS. The multivariate logistic regression model showed a predictive variability range of 69.5%-92.7%, with sensitivity indices of 98.6% for specificity, 96.7% for sensitivity, and 97.6% for accuracy.

Conclusion: The study found a significant association between poor OHS and adverse outcomes. Other factors in critically ill patients, such as the weekly application of chlorohexidine gluconate mouthwash and SOFA, significantly influenced this independent variable, which had a high propensity risk of 180.965. The optimal threshold for a weekly chlorhexidine mouthwash application was 15.5 times per week.

## Introduction

Oral hygiene is essential for the overall health of patients in intensive care units (ICU), as bacterial accumulation in the pharynx is linked to various systemic conditions, including cardiovascular disease, chronic obstructive pulmonary disease, endocarditis, and bacteremia [1]. Dental plaque is a complex milieu comprising microorganisms, their byproducts, and salivary secretions, facilitating microbial colonization. Pathogenic bacteria from dental plaque, when aspirated into the lower airways, may lead to respiratory and ventilator-associated pneumonia (VAP) [2].

Patients in the critical care unit frequently necessitate mechanical ventilation owing to compromised respiration resulting from trauma, medical ailments, or recent surgical procedures [3]. Oral health declines after admission, as endotracheal intubation and critical illness impair immunity, heighten the risk of xerostomia, and complicate access to oral care. Dental plaque accumulates swiftly in critically ill patients, resulting in colonization by microbial pathogens. Insufficient oral hygiene and extended mouth opening can intensify plaque accumulation [4].

Oral care is an essential component of nursing, enhancing oral health and expediting recovery by averting infections. Nurses are essential in delivering effective oral care, particularly in ICU [5]. Hospital policies advocate for thorough oral hygiene, encompassing daily brushing of teeth, gums, and tongue with a soft pediatric toothbrush, as well as moistening the oral mucosa and lips every four hours [6]. Ongoing nursing education and refresher training are crucial for recognizing hazardous practices and maintaining productivity. The oral care education program is an excellent initiative to ensure nurses' knowledge and skills remain current in bedside practice [7].

The Beck Oral Assessment Scale (BOAS) was used to assess the oral cavity and the effectiveness of oral care provided by nurses in collaboration with intensivists and dental specialists in the gum. However, this assessment system grades patients in terms of lip, gingival, oral mucosa, tongue, teeth, and saliva. It is suggested that nurses in ICU use this guide for a complete assessment of oral mucosa as a diagnostic tool. BOAS was used for oral hygiene assessment, which has a content validity with a correlation coefficient of 94% for dental plaque and 77% for gingivitis [8]. This score utilizes a grading scale from 1 to 4 and evaluates various evaluation regions, including the lips, gingiva, oral mucosa, tongue, and saliva. The evaluation is scored at 1 point each for smooth, pink, moist, and intact lips; gingival and oral mucosa; tongue; clean teeth devoid of debris; and abundant, thin, watery saliva. Two points are assigned for each of the following: somewhat dry red lips, pale dry isolated lesions on the gingiva and oral mucosa, dry prominent papillae on the tongue, minor debris on teeth, and an increase in saliva purulence. A score of 3 is designated for dry, swollen, isolated blisters on the lips, together with swollen, red gingiva and oral mucosa. Dry, swollen papillae with red lesions on the tongue, significant debris on the teeth, and sparse, thickened saliva. A score of 4 is assigned to critically ill patients with oedematous and inflamed lips, very dry, oedematous, and inflamed gingiva and oral mucosa, a highly dry, oedematous, engorged tongue with coating, teeth obscured by debris, and thick, mucid, viscid saliva. The aggregation of these BOAS assessment parameters results in a total BOAS value ranging from a minimum of 5 to a maximum of 20, with the following interpretative scores: 5 indicates no dysfunction, 6-10 indicates mild dysfunction, 11-15 indicates moderate dysfunction, and 16-20 indicates severe dysfunction [9].

Most institutions have adhered to the recommended protocol, formulated in conjunction with nursing, intensivists, and dental specialists, designed to facilitate increased moisture provision beyond conventional oral care. This protocol complies with standard oral care guidelines for both non-mechanically and mechanically ventilated critically ill patients, which mandate the application of 0.2% chlorhexidine mouthwash at least once daily and up to twice per nursing shift, depending on the patient's clinical conditions, medical documentation, and nursing staff expertise [10]. As previously stated, maintaining oral hygiene through tooth cleaning, brushing, and rinsing with chlorhexidine mouthwash as a component of daily oral care reduces the incidence of VAP in critically ill patients from 26% to 18%. Nonetheless, there is no evidence indicating an advantage regarding mortality, duration of mechanical ventilation, or length of ICU stay [11].

This study aims to examine the influence of inadequate oral health on adverse outcomes in critically ill patients, specifically targeting elevated mortality rates and extended hospitalizations in both mechanically and non-mechanically ventilated individuals while considering the dual effects of the potential confounders of the weekly administration of chlorhexidine gluconate mouthwash and the sequential organ failure assessment tool for critically ill patients.

## Materials and methods

A retrospective observational study was performed on 4,999 critically ill patients admitted to the ICU at the King Hussein Medical Centre (KHMC) within the Royal Medical Services in Amman, Jordan, from January 2018 to May 2022.

The critically ill patients included in this study were adults and the elderly who were admitted for a minimum of three consecutive days. Patients under 18 years of age were excluded from this study, and any patient with missing data for predefined tested variables exceeding 5% was also considered an exclusion criterion. This study encompassed both mechanically and non-mechanically ventilated patients, with the documented use of a mouthwash solution containing chlorhexidine gluconate as the primary active ingredient at least four times per week being an inclusion criterion. The concentration of chlorhexidine mouthwash utilized in this study was the standard 0.2% formulation available at our institution.

The registration number 9_8/2024, assigned to this study during the committee meeting on December 17, 2024, received approval from the institutional review board. A second and final approval for publication was secured from the technical and development directorate of our institution on January 15, 2025. The informed consent requirement from pediatric parents was waived due to the retrospective nature of this study, which was conducted in strict compliance with the standards established in the Helsinki ethical research protocols.

The data for this study were primarily acquired through a composite triad: the documented information in our institutional electronic recording system, Hakeem, the written notes from our intensivist and dental physicians, and the electronic and written documentation notes from critical care nursing. The available data primarily comprised two dimensions: independent variables either partially or exclusively associated with the critically ill patients under examination or independent variables pertinent to the oral health care protocol. The frequency of chlorohexidine mouthwash application in our institutional daily care within the admitted ICU was recorded as the number of documented applications per week, abbreviated as weekly mouthwash (WMW). This study utilized the BOAS as the objective tool to evaluate the oral health status (OHS) of admitted patients, categorizing OHS as better for BOAS scores ranging from 5 to 10 and as poorer for scores exceeding 10 and up to 20 points.

The dichotomous independent variables were categorized into binary groups based on the estimated overall cohort average threshold. The sequential organ failure assessment (SOFA) tool was classified into two categories: lower SOFA category (<7 points) and higher SOFA category (≥7 points). The length of stay (LOS) for the critically ill patients studied was categorized into shorter LOS (≤14 days) and longer LOS (>14 days). The anthropometric indices of the tested patients, as indicated by their calculated body mass indexes (BMIs), were categorized into lower obesity status (<28 kg/m^2^) and higher obesity status (≥28 kg/m^2^). The assessment baseline comorbidity burden, as indicated by the age-adjusted Charlson Comorbidity Index (AACCI), was categorized into lower comorbidity burden (AACCI <7) and higher comorbidity burden (AACCI ≥7). The study of critically ill patients was divided into two groups: non-elderly adults (aged <60 years) and the elderly cohort (aged ≥60 years). Non-specified independent variables, including gender, overall mortality, mechanical ventilation status, and levels of consciousness, were categorized into binary states: females and males, survivors and non-survivors, naïve and experienced in mechanical ventilation, and subjective lower and higher consciousness levels.

The principal adverse outcomes of interest, referred to as composite outcomes of interest (cOI), in this study included the occurrence of at least one of the following: mortality at any point during the admission, prolonged admission exceeding three weeks, unsuccessful extubation and weaning from mechanical ventilation lasting more than one day, a significant overall increase in the SOFA score throughout the admission period, and an escalation in deep sedation necessitating the use of muscle relaxants. The entire cohort examined in this study was primarily divided based on the adverse outcomes of interest into two categories: those with better adverse outcomes, referred to as better cOI and designated as Group I, and those with poorer adverse outcomes, termed poorer cOI and designated as Group II.

The chi-square analyses were performed on these two compared groups to reveal the distributional rate variations and the statistical significance of the independent variables of the tested patients while examining the corresponding odds ratios adjusted by their 95% confidence intervals, chi-square statistics, and the Pearson correlations for the dichotomized independent variables tested across the two adverse outcomes of interest related to Groups I and II.

This study initially examined the weekly administration of chlorhexidine mouthwash during critical care admissions as part of the oral health care protocol. This was analyzed using binary logistic regression to evaluate its correlation with the probability of adverse clinical outcomes versus better clinical outcomes. To address the inadequately adjusted association between the weekly use of mouthwash and negative outcomes, we conducted multiple logistic regression analyses to evaluate the relationship between the OHS of critically ill patients, categorized into better versus poorer OHS, and the percentage probability of cOI positivity, while controlling for two additional potential confounders: the WMW and the patients' critical illness risk, as indicated by their SOFA scores upon admission.

This multiple logistic regression analysis sought to elucidate the variability in predictions utilizing the Cox-Snell and Nagelkerke pseudo-R^2^ statistics, alongside sensitivity indices for specificity, sensitivity, and accuracy metrics; the significant coefficients of the regression and their directional influences; and the propensity risk for the likelihood of inferior cOI adjusted by their 95% confidence intervals. Additionally, we conducted a series of statistical tests, including binary logistic regression, receiver operating characteristic analysis, and sensitivity analyses, to determine the optimal threshold for the weekly applications of chlorhexidine mouthwash that corresponded with the highest composite values of sensitivity and specificity, as indicated by the maximum Youden's index value. Additionally, the area under the receiver operating characteristic curve was presented in this paper, along with its corresponding standard error and 95% confidence interval.

The principal tool for gathering, updating, and analyzing retrospective data in this study was Microsoft Excel (version 20; Microsoft® Corp., Redmond, WA). This study primarily utilized the Statistical Product and Service Solutions (SPSS, version 250; IBM SPSS Statistics for Windows, Armonk, NY) to statistically analyze the intended data. The threshold for statistical significance in this analysis was established at 5%.

## Results

A total of 4,999 critically ill patients participated in this retrospective study, with approximately 2,370 patients (47.41%) achieving improved cOI. Conversely, roughly 2,629 patients (52.26%) exhibited a positive result for at least one of the identified outcomes of interest and were consequently assigned to the inferior cOI group (Group II). The overall mortality rate in the poorer cOI group (Group II) was around 1,920 patients (73%), compared to the survival rate of roughly 709 patients (27%). Nearly all critically ill patients categorized within the poorer cOI cohort were mechanically ventilated, about 2,594 patients (98.7%), in contrast to roughly 35 patients (1.3%) who were not mechanically ventilated.

In this study, roughly 3,381 (667.6%) were evaluated as having a diminished level of consciousness, as indicated subjectively by the Glasgow Coma Scale, in contrast to around 1,618 patients (32.4%) with elevated levels of consciousness. This study identifies statistically significant variations in distributional rates between Groups I and II, demonstrating a moderate positive correlation between lower levels of consciousness in critically ill patients and poorer cOI (+0.655 ± 0.009), with an estimated unadjusted odds ratio of 54.06 (42.92-68.09). The projected ratios for higher consciousness to lower consciousness were disclosed as 1.83:1 and 0.03:1 for Group I and Group II, respectively.

The total number of examined females in this study was around 2,506 females (50.1%), while males constituted a slightly smaller percentage at about 2,493 males (49.9%), resulting in an estimated female-to-male ratio of 1.01:1. The distributional rate variations between Groups I and II among the tested genders were statistically significant, with estimated female-to-male ratios of 0.93:1 and 1.07:1, respectively, indicating a very weak negative correlation between being male and having a poorer cOI. The associated odds ratio was calculated as 0.868 (0.777-0.970).

In this study, elderly critically ill patients constituted roughly 1,665 patients (33.3%), while non-elderly patients accounted for about 3,334 patients (66.7%), resulting in an overall ratio of 2:1 for tested individuals aged below 60 years compared to those above. The distributional rate changes among cOI-related groups were statistically significant, with estimated ratios of 3.72:1 for non-elderly to elderly in Group I and 1.26:1 in Group II. The unadjusted Pearson correlation and estimated risk for aging in relation to poorer cOI were found to be +0.244 ± 0.013 and 2.952 (2.605-3.345), respectively.

This study demonstrated a statistically significant inverse correlation between obesity and diminished cOI in our critically ill patients (-0.101 ± 0.014). The obesity status of critically ill patients was determined using body mass indices, with a threshold selected that resembled the average BMI of all patients in this study, facilitating the distinction between lower and greater obesity statuses. The total odds ratio for inferior cOI in higher obese status compared to lower was calculated at 0.666 (0.596-0.745). The analysis demonstrated statistically significant variations in distribution rates between Group I and Group II (ꭓ2=50.851, p-value=0.000), with approximately 58.3% (1,382 patients) in Group I and approximately 48.2% (1,268 patients) in Group II exhibiting a higher BMI (≥28 kg/m^2^). The overall estimated ratio for lower obesity (<28 kg/m^2^) compared to higher obesity (≥28 kg/m^2^) was 0.89:1.

Prognostically, all critically ill patients in this study with a superior cOI (Group I) had an AACCI of <7 points, whereas all critically ill patients classified in the inferior cOI group (Group II) had an AACCI of ≥7 points. Nevertheless, the overall ratio of the burden of lower critically ill patients to that of higher comorbidity load was identified as 0.90:1. The SOFA score, categorized into lower scores (<7 points) and higher scores (≥7 points), exhibited a statistically significant variation in distribution rates between superior and inferior cOI groups. A robust positive correlation was observed between elevated SOFA scores and diminished cOI in our critically ill patient cohort (+0.805 ± 0.008), with an estimated odds ratio of approximately 183.69 (139.41-242.04). The estimated ratio of critically ill patients with lower SOFA scores (<7 total points) to those with higher SOFA scores (≥7 total points) was calculated to be 0.657:1. The distribution percentages for greater SOFA scores in comparison to lower SOFA scores within the superior cOI group (Group I) were 448 patients (18.9%) and 1922 critically ill patients (81.1%), respectively.

The length of stay (LOS) for the critically ill patients evaluated demonstrated a statistically significant difference, with an overall estimated ratio of shorter LOS (<14 days) to longer LOS (≥14 days) found as 1.13:1. All patients with a better cOI exhibited a length of stay of no less than 14 days, with no reported early mortality in this study (mortality occurring prior to the completion of 14 days). The robust link and minimal odds ratio of discharge prior to two weeks post-ICU admission underscored the significance of minimizing the risk of adverse cOI among the critically ill patients evaluated in this study: -0.992 ± 0.002 and 0.008 (0.005-0.012).

Upon evaluating the statistical outcomes for the independent variables associated with the oral health status of critically ill patients, we identified a robust positive correlation between the BOAS (5-20) and the dichotomized subjective oral health statuses (OHS) of superior versus inferior OHS, with higher BOAS scores correlating with poorer OHS and a cOI of +0.953 ± 0.004 and +0.900 ± 0.003, respectively. Nevertheless, the estimated odds ratio for the patients with OHS was reported as 2,160.4 (1434.2-3254.5). The variations in distributional rates for both examined oral health-related independent variables across the patients' outcomes of interest (better cOI versus poorer cOI groups) demonstrated statistical significance. The evaluated patients' BOAS overall scores in this study were classified into sequential ascending ranges: the 5 total scoring category, the 6-10 category, the 11-15 category, and finally the 16-20 category. The distribution rates for these BOAS scores were 1,435 (28.7%), 976 (19.5%), 783 (15.7%), and 1,805 (36.1%). The overall estimated ratio of superior to inferior OHS was determined to be 0.94:1, with an estimated ratio of 73.1:1 for superior cOI and 0.034:1 for inferior cOI, respectively. The chi-square analysis results are fully expressed in Tables 1-2.

**Table 1:** Chi-square analysis results (part 1). The chi-square analyses were performed on these two compared groups to reveal the distributional rate variations and the statistical significance of the independent variables of the tested patients while examining the corresponding odds ratios adjusted by their 95% confidence intervals, chi-square statistics, and the Pearson correlations for the dichotomized independent variables tested across the two adverse outcomes of interest related to Groups I and II. The chi-square statistical test was employed in this study to extract the corresponding p-values, indicating the significance of the variables of the studied patients across the two investigated groups, Group I and Group II. Group I: Cohort with better (superior) composited outcomes of interest. Group II: Cohort with poorer (inferior) composited outcomes of interest. OR: Odds ratio; CI: Confidence interval; LL: Lower limit; UL: Upper limit; x^2^: Chi statistic; p-value: Significance level of 5%; R: Pearson correlation; SEV: Standard error of value; MOR: Mortality; MV: Mechanical ventilation; r: Ratio; F: Female; M: Male; Yrs: Years; BMI: Body mass index; Kg: Kilogram; m²: Square meter; NA: Statistically not applicable; *: Statistically significant (p-value < 0.05)

	Better cOI	Poorer cOI	Overall Cohort	OR (CI; LL-UL)	R±SEV	x^2^	p-value
	Group I	Group II					
	2370, 47.41%	2629, 52.26%	4999				
MOR Status							
Survivors	2370 (100.0%)	709 (27.0%)	3079 (61.6%)	0.230 (0.216-0.246)	+0.750±0.008	2810.2	0.000*
Non-Survivors	0 (0.0%)	1920 (73.0%)	1920 (38.4%)				
r	NA	0.37:1	1.6:1				
MV							
Naive	2369 (100.0%)	35 (1.3%)	2404 (48.1%)	175577 (24035-1282572)	+0.986±0.002	4856.658	0.000*
Experienced	1 (0.0%)	2594 (98.7%)	2595 (51.9%)				
r	2369: 1	0.01: 1	0.93: 1				
Gender							
F	1144 (48.3%)	1362 (51.8%)	2506 (50.1%)	0.868 (0.777-0.970)	-0.035±0.014	6.236	0.013*
M	1226 (51.7%)	1267 (48.2%)	2493 (49.9%)				
r	0.93:1	1.07:1	1.01:1				
Age (Yrs)							
<60	1868 (78.8%)	1466 (55.8%)	3334 (66.7%)	2.952 (2.605-3.345)	+0.244±0.013	298.268	0.000*
≥60	502 (21.2%)	1163 (44.2%)	1665 (33.3%)				
r	3.72:1	1.26:1	2.00:1				
BMI (kg/m^2^)							
<28	988 (41.7%)	1361 (51.8%)	2349 (47.0%)	0.666 (0.596-0.745)	-0.101±0.014	50.851	0.000*
≥28	1382 (58.3%)	1268 (48.2%)	2650 (53.0%)				
r	0.71:1	1.07:1	0.89:1				

**Table 2:** Chi-square analysis results (part 2). The chi-square analyses were performed on these two compared groups to reveal the distributional rate variations and the statistical significance of the independent variables of the tested patients while examining the corresponding odds ratios adjusted by their 95% confidence intervals, chi-square statistics, and the Pearson correlations for the dichotomized independent variables tested across the two adverse outcomes of interest related to Groups I and II. The chi-square statistical test was employed in this study to extract the corresponding p-values, indicating the significance of the variables of the studied patients across the two investigated groups, Group I and Group II. Group I: Cohort with better (superior) composited outcomes of interest. Group II: Cohort with poorer (inferior) composited outcomes of interest. OR: Odds ratio; CI: Confidence interval; LL: Lower limit; UL: Upper limit; x^2^: Chi statistics; p-value: Significance level of 5%; R: Pearson correlation; SEV: Standard error of value; r: Ratio; AACCI: Age-adjusted Charlson comorbidity index; LOC: Level of consciousness; BOAS: Beck Oral Assessment Scale (5-20); NA: Statistically not applicable; OHS: Oral health status; LOS: Length of stay; SOFA: Sequential organ failure assessment; *: Statistically significant (p-value < 0.05)

	Better cOI	Poorer cOI	Overall Cohort	OR (CI; LL-UL)	R±SEV	x^2^	p-value
	Group I	Group II					
	2370, 47.41%	2629, 52.26%	4999				
AACCI							
<7	2370 (100.0%)	0 (0.0%)	2370 (47.4%)	NA	NA	4999	0.000*
≥7	0 (0.0%)	2629 (100.0%)	2629 (52.6%)				
r	NA	NA	0.90:1				
LOC							
Higher	1532 (64.6%)	86 (3.3%)	1618 (32.4%)	54.06 (42.92-68.09)	+0.655±0.009	2144.4	0.000*
Lower	838 (35.4%)	2543 (96.7%)	3381 (67.6%)				
r	1.83:1	0.03:1	0.48:1				
BOAS							
5	1425 (60.1%)	10 (0.4%)	1435 (28.7%)	NA	+0.900±0.003	4528.868	0.000*
6-10	903 (38.1%)	73 (2.8%)	976 (19.5%)				
11-15	42 (1.8%)	741 (28.2%)	783 (15.7%)				
16-20	0 (0.0%)	1805 (68.7%)	1805 (36.1%)				
OHS							
Better	2338 (98.6%)	86 (3.3%)	2424 (48.5%)	2160.4 (1434.2-3254.5)	+0.953±0.004	4539.562	0.000*
Poorer	32 (1.4%)	2543 (96.7%)	2575 (51.5%)				
r	73.1:1	0.034:1	0.94:1				
LOS (d)							
>14	20 (0.8%)	2629 (100.0%)	2649 (53.0%)	0.008 (0.005-0.012)	-0.992±0.002	4919.390	0.000*
≤14	2350 (99.2%)	0 (0.0%)	2350 (47.0%)				
r	0.009:1	NA	1.13:1				
SOFA							
<7	1922 (81.1%)	60 (2.3%)	1982 (39.6%)	183.69 (139.41-242.04)	+0.805±0.008	3235.629	0.000*
≥7	448 (18.9%)	2569 (97.7%)	3017 (60.4%)				
r	4.29:1	0.023:1	0.657:1				

This study did multiple logistic regression analysis to predict the likelihood of lower cOI compared to better cOI in a cohort of 4,999 critically sick patients, with reported mean ± SD values for age, length of stay, body mass index, AACCI, SOFA, ventilation-free days, BOAS, and the weekly application of mouthwash times assessed at 48.93 ± 17.412 years, 15.11 ± 8.259 days, 28.124 ± 6.665 kg/m², 7.04 ± 3.785, 7.04 ± 2.294, 0.67 ± 1.362 days, 2.59 ± 1.240, and 14.08 ± 4.898, respectively. The multiple logistic regression analysis conducted revealed a statistically significant relationship among the three independent variables: the primary independent variable of admitted patients' OHS, comparing better OHS to poorer OHS, alongside the other two contributing factors, namely, the frequency of weekly mouthwash applications (WMW) and the SOFA scores of critically ill patients.

The regression association for the OHS relative to the probability of poorer cOI in this study was positively expressed at 5.198 ± 0.340, indicating a higher risk for poorer OHS compared to better OHS, with a propensity risk of 180.965 (92.865-352.645). The regression analysis of the patients' evaluated SOFA scores indicated a positive association of 1.740 ± 0.223 for each additional SOFA point, with a propensity risk of 5.696 (3.682-8.811). This study demonstrates a statistically significant positive regression association with the frequency of chlorhexidine mouthwash applications per week, exhibiting a regression coefficient of 0.196 ± 0.043 for each additional application and a propensity risk of 1.216 (1.117-1.324). The multiple logistic regression analysis was established as follows:

% Probability for cOI positivity = e (5.997 + 1.740 SOFA + 0.196 WMW + 5.198 OHS)/1 + e (5.997 + 1.740 SOFA + 0.196 WMW +5.198 OHS)

This multiple logistic regression model exhibited a predictive variability range of 69.5%-92.7%, as indicated by the Cox-Snell and Nagelkerke pseudo-R^2^ statistics, and demonstrated a significant chi-square statistic of ꭓ²(7)=97.788, p-value=0.000. The sensitivity indices for the developed multiple logistic regression model were recorded at 98.6% for specificity, 96.7% for sensitivity, and 97.6% for accuracy. The results of the various logistic regression analyses are summarized in Table 3.

**Table 3:** Multiple logistic regression analysis outcomes. To address the inadequately adjusted association between the weekly use of mouthwash and negative outcomes, we conducted multiple logistic regression analyses to evaluate the relationship between the oral health statuses (OHS) of critically ill patients, categorized into better versus poorer OHS, the percentage probability of cOI positivity, and controlling for two additional potential confounders: the WMW and the patients' critical illness risk, as indicated by their SOFA scores upon admission. B: Regressional coefficient; SE: Standard error; Sig: Statistically significant; Exp (B): Exponent or propensity risk; CI: Confidence interval; LL: Lower limit; UL: Upper limit; VR: Variability range; x^2^: Chi statistic; df: Degree of freedom; p-value: Statistically significant at a threshold of 5%; TNR: True negative rate or specificity; TPR: True positive rate or sensitivity; AI: Accuracy index; Prob: Probability; cOI: Composited outcomes of interest; OHS: Oral health status; WMW: Weekly application frequency for the chlorhexidine gluconate-based mouthwash; SOFA: Sequential organ failure assessment

Items	B±SE	Sig	Exp (B) (95% CI; LL-UL)	%VR	p-value
%Prob cOI positivity					
OHS	5.198±0.340	0.000*	180.965 (92.87-352.645	69.5%-92.7%	0.000
WMW	0.196±0.043	0.000*	1.216 (1.117-1.324)		
SOFA	1.740±0.223	0.000*	5.696 (3.682-8.811)		
Constant	-5.997±0.486	0.000*	0.002		

**Figure 1:**
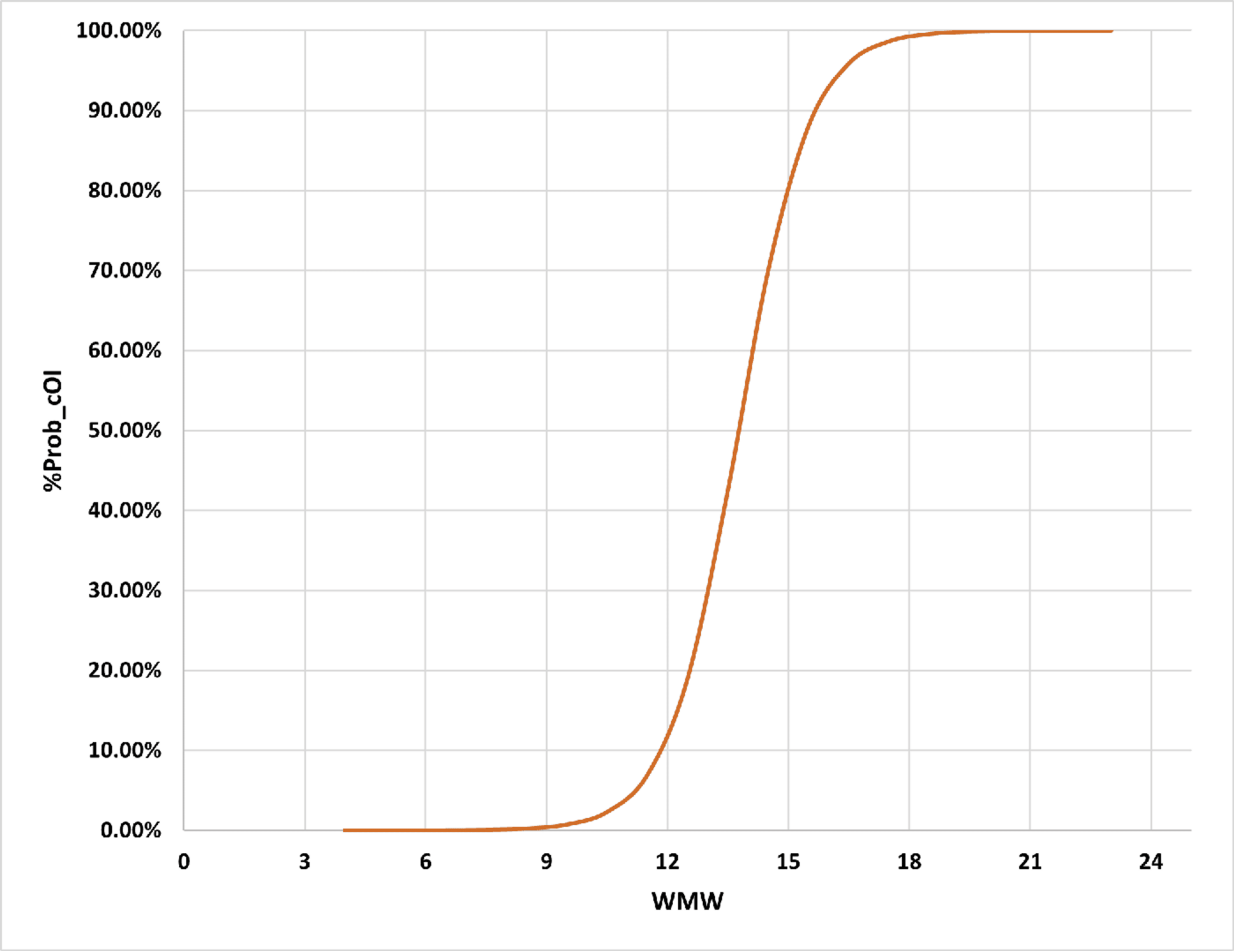
Multiple logistic regression analysis outcomes. To address the inadequately adjusted association between the weekly use of mouthwash and negative outcomes, we conducted multiple logistic regression analyses to evaluate the relationship between the oral health statuses (OHS) of critically ill patients, categorized into better versus poorer OHS, and the percentage probability of cOI positivity while controlling for two additional potential confounders: the WMW and the patients' critical illness risk, as indicated by their SOFA scores upon admission. B: Regressional coefficient; SE: Standard error; Sig: Statistically significant; Exp (B): Exponent or propensity risk; CI: Confidence interval; LL: Lower limit; UL: Upper limit; VR: Variability range; x2: Chi statistic; df: Degree of freedom; p-value: Statistically significant at a threshold of 5%; TNR: True negative rate or specificity; TPR: True positive rate or sensitivity; AI: Accuracy index; Prob: Probability; cOI: Composited outcomes of interest; OHS: Oral health status; WMW: Weekly application frequency for the chlorhexidine gluconate-based mouthwash; SOFA: Sequential organ failure assessment

To underscore the correlations between the weekly application of chlorhexidine gluconate mouthwash in critical care oral health management, we performed sequential receiver operating characteristics, binary logistic regression, and sensitivity analyses. This study determined the optimal weekly application to be 15.5 times, yielding sensitivity indices of 82.00% for sensitivity, 89.10% for specificity, 84.16% for accuracy, and 95.27% for both positive and negative predictive values. The area under the ROC curve (AUROC) was calculated as 0.962 ± 0.003 (95% CI; LL-UL: 0.957-0.967). The binary regression analysis depicting the relationship between weekly applications of chlorhexidine mouthwash and the likelihood of unfavorable adverse outcomes is presented in Figure 1.

## Discussion

This study was designed as a retrospective observational analysis conducted in the ICU at the Royal Medical Services for admitted Jordanian critically ill patients, with a total eligible sample size of 4,999 patients, both mechanically ventilated and non-mechanically ventilated, during the period from January 2018 to May 2022. This study primarily investigated the occurrence, as a probability function, of at least one of the predefined adverse outcomes of interest, including but not limited to admission duration exceeding 21 days, failure to extubate for more than one day, persistent uptrending in the SOFA scale, and overall mortality. The negative outcomes of interest were collectively referred to as cOI. The occurrence of at least one of these examined adverse outcomes was deemed indicative of positivity for cOI.

Nonetheless, the probability of cOI positivity (a positive state) was associated with a proposed independent variable selected for examination in this study. Mechanistically, these prognostic indicators were propped to predict the likelihood of patients experiencing adverse outcomes of interest, including the primary prognostic factor examined in this study, the patient's OHS, alongside the frequency of weekly application of 0.2% chlorhexidine gluconate mouthwash and the assessment of risk in critically ill patients as indicated by their SOFA. Our study's uniqueness lies in its inclusion of both mechanically and non-mechanically ventilated patients, thereby enhancing the generalisability of the findings. The study uniquely investigates the prognostic impact of patients' OHS while considering dual potential confounders that underscore the quality of intensive care protocol implementations. This involves daily oral care for patients using a 0.2% chlorhexidine gluconate mouthwash, measured by the total number of applications per week per patient, and evaluating the overall risk of adverse outcomes as indicated by the assessment of patients' SOFA scores.

This study's uniqueness was underscored by our investigation into the optimal weekly application frequency of chlorhexidine mouthwash for standard critically ill patients, aiming to achieve the highest balance between sensitivity and specificity regarding the mouthwash's impact on adverse outcomes in this population. Furthermore, another distinctive aspect of this study was the implementation of an objective-based assessment tool for evaluating the OHS of critically ill patients, as opposed to relying predominantly on subjective research methods. This study utilized the BOAS to categorize the overall tested cohort into two OHS categories: better OHS with BOAS scores ranging from 5 to 10 and poorer OHS with summed BOAS scores between 10 and 20. This may provide our multiple logistic regression analyses results and interpretations of this study with an advantage.

Indeed, poor oral health is linked to poor systemic health, making oral hygiene crucial in preventing healthcare-associated infections and contributing to mortality among critically ill patients [12]. Older oral care practices in ICUs vary due to factors such as equipment availability, patient dentist status, and healthcare providers' knowledge and experience. Several oral hygiene interventions have been integrated into specific protocols to prevent VAP in critically ill patients, but there is limited evidence of efficacy [13]. Challenges in implementing oral hygiene protocols include lack of time, inadequate staffing, lack of evidence supporting oral care, unawareness, no training, nursing staff shortage, limited oral care knowledge, and no oral care priorities and competencies [14]. Product manufacturers have developed preventive hygiene products for ventilated patients, such as specially shaped toothbrushes or reservoirs attached to a toothbrush. Suction devices have also been developed for mechanically ventilated patients in ICU. Digital tools and telemedicine in managing oral care are being discussed in the professional care community [15].

Most prior studies and literature reviews assessing the impact of oral hygiene protocols on dental plaque and gum indices in critically ill patients admitted to the ICU employed specific inclusion and exclusion criteria, particularly age restrictions, and excluded critically ill patients with coagulopathy or those receiving therapeutic anticoagulant dosing, which is impractical in actual ICU accommodating critically ill patients [16]. Moreover, the majority of these studies employed a fixed dosing frequency of chlorhexidine, which is seldom observed in clinical practice due to the significant variability in the conditions of critically ill patients admitted. Consequently, most of these studies involve a relatively small number of eligible critically ill patients for examination [17]. In our study, we limit the exclusion and inclusion criteria to accurately reflect the acute nature and variability of the statuses of ICU-admitted patients, thereby creating a study that closely simulates real-world clinical practice. This study encompassed a substantial sample size of 4,999 patients admitted to our ICU department over a duration of four and a half years. To account for the inherent variability and multidimensional potential confounders that may influence the primary clinical outcomes of admitted ICU patients, we chose to adjust our regression analyses of the patient's OHS and adverse outcomes of interest by incorporating the patients' evaluated SOFA tool.

A study by Zhao et al. examined the impact of OHC on the likelihood of adverse outcomes, particularly the incidence of VAP, in mechanically ventilated critically ill patients. This study included 40 patients and was conducted as a randomized prospective controlled trial. The oral healthcare examined in this study primarily included pediatric toothbrushes, rinses, mouthwash, and gels, or a combination of some of these. The authors concluded that chlorhexidine, incorporated into the modified oral care protocols for critically ill patients in this randomized study, was associated with a significant reduction in the incidence of VAP in mechanically ventilated patients, decreasing the risk from 26.5% to 18% [18].

Another randomized controlled prospective study was conducted by de Lacerda Vidal et al. on 716 patients, with 108 randomized to the control and 105 to the intervention groups. This study aimed to investigate the role of 0.12% chlorhexidine mouthwash combined with pediatric tooth brushing as part of the oral hygiene regimen in mechanically ventilated patients to assess the impact of this dual approach on reducing the incidence of VAP. Nonetheless, the authors concluded that the implementation of a dual mouthwash (0.12%) in conjunction with toothbrushing significantly reduced the risk of acquiring VAP and decreased the overall prevalence of VAP during the study period. However, they did not determine that there was a statistically significant difference in the variations concerning admissions to the ICU. They recommend implementing at least one of these dual oral healthcare strategies for critically ill patients to reduce the prevalence and incidence of VAP in those admitted to the ICU [19].

Analogous studies, including randomized trials and systematic reviews/meta-analyses, were identified in the literature. A systematic study by Shi et al. evaluated the clinical impacts and efficacy of various chlorhexidine-based impregnated solutions, including mouthwashes, rinses, gels, and pastes, with or without adjunctive toothbrushing, primarily in pediatric populations. The study assessed the overall prevalence of new incidences of VAP in mechanically ventilated, critically ill patients and found a statistically significant reduction in the risk and incidence of VAP in adult patients admitted to the ICU. This systematic review selected eight randomized controlled trials, concluding that chlorhexidine, employed through various vehicles including mouthwash applications, significantly reduced the risk of VAP by an average of 36% compared to the control groups in the included trials. Nevertheless, the chlorhexidine concentration utilized in the trials included in this systematic review was approximately 2%. Indeed, these clinical trials included randomized studies; even though the variation across these studies was considered low, it did not reveal a statistically significant benefit on mortality rates [20].

The final study referenced in our research was conducted by Steinle et al., who highlighted the investigation of clinical benefits and positive outcomes associated with improved oral health in critically ill patients admitted to the ICU. Our study results predominantly align with those of Steinle et al., which demonstrated positive clinical impacts, or conversely stated, a reduced propensity for adverse outcomes concerning overall mortality and LOS. Nonetheless, Steinle et al. also reported a statistically significant improvement in medication burden, infectious burden, and specifically in end-stage renal failure [21].

A study by Alotaibi et al. assessed the effect of oral care guidelines on the oral care provided to mechanically ventilated patients by ICU nurses. The study involved 215 enrolled nurses, and demographic data along with oral care practices were collected via a self-administered survey. The results indicated that participants adhering to oral care recommendations exhibited markedly superior oral care practice scores compared to those lacking such guidelines, implying that oral care protocols may mitigate morbidity and death associated with VAP [22].

Our study revealed that 47.41% achieved enhanced cOI, whereas 52.26% were categorized in the inferior cOI group. The overall mortality rate in the lower socioeconomic group was 73%, in contrast to a survival rate of 27%. The majority of patients were subjected to mechanical ventilation, with 98.7% receiving this intervention. The LOS for critically ill patients exhibited a notable disparity, with a shorter LOS (<14 days) in contrast to a longer LOS (≥14 days). The study identified a robust positive correlation between the BOAS (5-20) and subjective OHS, indicating that higher BOAS scores and poorer OHS are associated with an increased likelihood of adverse outcomes and inferior clinical oral indicators (cOI). It is important to note that our study identified an optimal threshold of chlorhexidine gluconate application at 15.5 times per week, which was linked to the best clinical outcomes. Additionally, a significant positive correlation was observed with the frequency of chlorhexidine mouthwash applications per week.

This study was methodologically limited by its retrospective observational design and its execution at a single center, despite the Royal Medical Services being regarded as a multi-institutional network across Jordan. The study was specifically conducted in the ICU of the King Hussein Medical Centre in Amman. Nonetheless, our study highlights a comparatively adequate sample. Establishing causation is preferable to mere association through the implementation of prospective randomized controlled trials.

## Conclusions

This study demonstrated the significance of OHS in critically ill patients admitted to medical facilities, encompassing both mechanically and non-mechanically ventilated individuals, in relation to their propensity for adverse outcomes, including prolonged ICU stay and elevated mortality rates. Consequently, it is recommended that the OHS of critically ill patients be evaluated using a more systematic approach, such as the BOAS employed in this study. However, it appears that the OHS of admitted critically ill patients still exhibited a statistically significant correlation with poorer outcomes, despite accounting for the contributory regression associations related to the use of chlorhexidine mouthwash as the standard protocol for oral decontamination in our institution and other global ICUs. The significant effects of the admitted patient risk tool, particularly the SOFA, as utilized in this study. We recommend incorporating daily assessments of the OHS of critically ill patients and administering chlorhexidine mouthwash at least 15 times per week as part of oral health care. Dental professionals can substantially impact the decisions made by healthcare practitioners, and the provision of early, suitable, and ongoing education and training for all healthcare professionals involved in patient care is the sole path forward.
